# A Sincere Thank You to Our 2021 Peer Reviewers

**DOI:** 10.31486/toj.21.5022

**Published:** 2021

**Authors:** Ronald G. Amedee

**Affiliations:** Head of Clinical School and Professor, The University of Queensland Faculty of Medicine, Ochsner Clinical School, New Orleans, LA; Editor-in-Chief, *Ochsner Journal*

*There is no winter without snow, no spring without sunshine, and no happiness without companions.*

*–Korean proverb*

Nine original research articles, one systematic review, one quarterly column, and eight case reports and clinical observations are the principal elements of the Winter 2021 issue of the *Ochsner Journal*. As featured on the cover of this issue, we have a mini-COVID-19 theme reflected in two original research articles and two case reports. “Mental Health Appointments in the Era of COVID-19: Experiences of Patients and Providers” by Hunsinger, Hammarlund, and Crapanzano describes essential clinical contingency planning implemented to facilitate patient care during the pandemic. “Observational Study of the Clinical Characteristics and Short-Term Outcomes of Kidney Transplant Recipients Diagnosed With COVID-19 Infection (SARS-CoV-2) Requiring Hospitalization in New Orleans” by Giusti, Chazin, Vaitla, et al is a particularly enlightening manuscript to review at our current point in the COVID-19 pandemic recovery. One of the coauthors of this paper, Dr Jorge Garces, is the featured contributor for the winter issue. His impressive bio is posted at the *Journal* website (www.ochsnerjournal.org). The COVID-19–related case reports are “De Novo Immunoglobulin A Vasculitis Following Exposure to SARS-CoV-2 Immunization” submitted by Mohamed, Wickman, Fogo, and Velez and “Multicentric Breast Abscesses in a Patient Who Had COVID-19” by Van Wert, Ghio, Graham, et al.

The topics of our other original research articles range from an analysis of the effect of antiplatelet and anticoagulant agents on total joint arthroplasty outcomes to identifying patients who are good candidates for left ventricular assist device removal.

Pastrak, Abd-Elsayed, Ma, et al have provided a “Systematic Review of the Use of Intravenous Ketamine for Fibromyalgia.” Case report subjects include hypersensitivity pneumonitis, primary pulmonary pleomorphic liposarcoma, heterotropic triplet pregnancy, and anaphylactic reactions to isosulfan blue dye. Our quarterly Sports Medicine column examines the “Utility of Percutaneous Needle Tenotomy to Reduce Pain and Improve Function in Common Extensor Tendinosis of the Lateral Epicondyle” and was authored by Hatamiya, Kobayashi, and Gottschalk.

As we publish this final issue of 2021, it is impossible to forget the heroic efforts of our leaders, faculty, staff, and learners at Ochsner Health which allowed us to successfully emerge from a third and fourth surge of COVID-19 along with overcoming a major natural disaster in our state/region called Hurricane Ida. The “Power of One” was dramatically displayed during these periods of crisis. Thank you all for everything you do for our patients and health care organization each day. Nearly 3.1 million doses of vaccine have been administered in our state, and presently 2.26 million Louisiana citizens are fully vaccinated. However, work remains in our vaccination efforts as Louisiana has more than 4.9 million citizens. As I write this introduction, we are experiencing growing concerns about the Omicron COVID-19 variant. It is too soon to tell whether this new strain will result in yet a fifth surge for us to address in the coming weeks.

Fall has finally arrived in New Orleans, and winter is rapidly headed our way, but this current season at least marks the end of a very active hurricane season. In Louisiana, we do experience winter but usually without snow. We are blessed with many days of sunshine all throughout the year.

As we close our 2021 publication year, it is important to once again thank all reviewers who submitted timely and constructive reviews, the backbone of any peer-reviewed journal. We could not publish without your help and are sincerely grateful for every thoughtful comment. Your work makes our work better. A special thanks also goes to the editorial staff led by Managing Editor Kathleen McFadden and Camille Barnett who serves as medical editor.

On behalf of the editorial staff, the *Ochsner Journal* Editorial Board, and the Division of Academics at Ochsner Health, I wish all our readers Happy Holidays and a prosperous New Year 2022.

